# Functional groups in piscivorous fishes

**DOI:** 10.1002/ece3.8020

**Published:** 2021-09-02

**Authors:** Michalis Mihalitsis, David R. Bellwood

**Affiliations:** ^1^ Research Hub for Coral Reef Ecosystem Functions James Cook University Townsville Qld Australia; ^2^ College of Science and Engineering James Cook University Townsville Qld Australia; ^3^ Australian Research Council Centre of Excellence for Coral Reef Studies James Cook University Townsville Qld Australia

**Keywords:** capture, engulfer, grabber, predation, prey size, strike

## Abstract

Piscivory is a key ecological function in aquatic ecosystems, mediating energy flow within trophic networks. However, our understanding of the nature of piscivory is limited; we currently lack an empirical assessment of the dynamics of prey capture and how this differs between piscivores. We therefore conducted aquarium‐based performance experiments, to test the feeding abilities of 19 piscivorous fish species. We quantified their feeding morphology, striking, capturing, and processing behavior. We identify two major functional groups: grabbers and engulfers. Grabbers are characterized by horizontal, long‐distance strikes, capturing their prey tailfirst and subsequently processing their prey using their oral jaw teeth. Engulfers strike from short distances, from high angles above or below their prey, engulfing their prey and swallowing their prey whole. Based on a meta‐analysis of 2,209 published in situ predator–prey relationships in marine and freshwater aquatic environments, we show resource partitioning between grabbers and engulfers. Our results provide a functional classification for piscivorous fishes delineating patterns, which transcend habitats, that may help explain size structures in fish communities.

## Graphical abstract

We identify functional groups of piscivorous fishes based on their ability to strike at, capture, and process prey. Also, we show that these groups reflect resource partitioning in aquatic habitats.

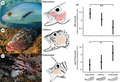

## 1 INTRODUCTION

Predation is a fundamental process in all ecosystems. It is a key process through which energy and nutrients are transported between organisms. Humans have been aware of this process since the Pleistocene, when early hominin species were still part of the food chain (Berger, 2006; Brantingham, 1998; Treves & Palmqvist, 2007). In many respects, terrestrial predation is part of human evolution. Yet, aquatic predation has been present for considerably longer and is likely to strongly shape the life history of aquatic animals. While humans have been aware of aquatic fauna for millennia (Elkin, 1952), it is only in the last few decades that technology has allowed humanity to unravel its mechanistic basis and to quantify its impact on ecosystems.

Today, almost every aquatic ecosystem has been examined with regard to predation. For example, multiple studies have demonstrated the ability of predators in upper trophic levels (i.e., fishes) to influence food webs through top‐down control (Carpenter et al., 2001; Hansson et al., 2007; Jeppesen et al., 2003). Nevertheless, while this concept has been found to operate in relatively simple ecosystems, such as lakes, recent work in more diverse aquatic ecosystems have not found similar patterns (Casey et al., 2017; Desbiens et al., 2021; Grubbs et al., 2016; Malakhoff & Miller, 2021; Rizzari et al., 2015; Roff et al., 2016). Part of this may be the complexity (i.e., functional diversity) of the predators. Therefore, there may be a need to first establish how piscivores influence their prey (i.e., the exact niche axis on which their function is expressed), before attempting to scale up potential effects at an ecosystem level.

Previous work has shown that different “types” of predators (Hobson, 1979; Juanes et al., 2002) can have different influences on communities (Hixon & Carr, 1997). This becomes particularly relevant given the taxonomically heterogenous nature of predator assemblages within different habitats and ecosystems (Burress et al., 2013; Mihalitsis & Bellwood, 2019a; Winemiller, 1989), not only due to biogeography (Hemingson & Bellwood, 2018) but also due to direct anthropogenetic impacts (e.g., overfishing, invasive species) (Albins & Hixon, 2008; Graham et al., 2005; Green & Côté, 2014; Valdivia et al., 2017). Yet, we know little of the ecological impacts of these heterogenous predator assemblages. Do they deliver different types of predation on the communities they live in? In essence, there is a need to understand the different types of predators in aquatic ecosystems, the effect of each predator type on its prey and, ultimately, on its community and ecosystem in general (i.e., functional groups sensu Bellwood et al. 2019).

To date, multiple studies have described different “types” of piscivorous fishes (i.e., fish feeding on fish). Hobson (1979) described four major behaviors of piscivores with regard to prey capturing, namely (1) running down prey, (2) ambushing, (3) habituating prey to an illusion that they are nonaggressive, and (4) stalking. Hixon and Carr (1997) further classified piscivores as “resident” or “transient,” based on whether the predator inhabits the same habitat as its prey or regularly swims between habitats. Indeed, there is a wide range of terms from ambush and sit‐and‐wait, to pursuit. By searching the literature, we found a total of 10 different terms in common use, mostly based on behavior with the same species often having multiple classifications (Table S1). More recently, Mihalitsis and Bellwood (2019a) identified three major ecomorphotypes of piscivores: diurnal benthic, nocturnal, and pelagic, while Mihalitsis and Bellwood (2019b) identified three distinct morphotypes, based on their dentition and feeding traits: edentulate, villiform, and macrodont morphotypes. Essentially, there appear to be major differences between piscivorous fishes, suggesting high within‐group variation in feeding capabilities and behaviors. However, this raises the question: Do these different predator types also reflect differences in their feeding performance, behavior, and, ultimately, their impact on associated ecosystems?

The goal of this study, therefore, is to quantify aquatic predation by piscivorous fishes through performance‐based feeding experiments. Using these data, we explore their potential impact on prey populations/communities, placing their functional abilities in an ecological context, through a meta‐analysis of relative prey sizes found in piscivorous fishes from multiple aquatic habitats.

## 2 MATERIALS AND METHODS

We conducted performance‐based feeding experiments to assess the implications of morphological variation on the performance of piscivorous fishes when capturing and ingesting prey. Feeding events were filmed, and the videos were analyzed to extract quantitative measurements of the approach, strike capture, and subsequent handling of prey. We used piscivorous coral reef fishes as a study group.

### 2.1 Performance experiments

Performance experiments were carried out in a climate‐controlled room (27°C), between 2018 and 2021 at James Cook University (JCU). Housing and experimental protocols were in accordance with the JCU Animal Ethics Committee (A2523). Holding and experimental tanks were connected in a flow‐through filtration system, with halogen lighting above tanks between 9 a.m. and 6 p.m. When not in experimental trials, prey fish were fed commercially available flake and pellet food, while predators were fed commercially available pieces of prawn. We used predators of all three benthic‐associated morphotypes: edentulate, villiform, and macrodont (sensu Mihalitsis and Bellwood 2019b), from a range of different families. We used a minimum of three different predator species within each morphotype, 1–4 individuals of each predator species (depending on availability), and for each individual, we recorded a minimum of 3 feeding events (range 3–10). Predator body sizes ranged from 51 mm standard length (SL) to 290 mm SL. In total, we examined 32 fish from 19 species, encompassing the majority of piscivorous coral reef fish families (Mihalitsis & Bellwood, 2019a). Experiments were carried out in 20L aquaria for small‐sized or “sit‐and‐wait” predators, and 120L aquaria for large‐bodied or more “active” predators. Only one predator was held in an aquarium at a time and was acclimatized for at least one week prior to experiment initiation.

Predators were starved for 24 hr prior to experimental feeding. Prior to experimentation, an opaque tank separator divided the tank into two arenas, to ensure predator and prey could not see each other. A single prey fish (*Acanthochromis polyacanthus*) was then measured for its SL and body depth (BD) in a zip‐lock bag (to avoid skin contact and to prevent potential effects of handling on predator behavior due to olfactory cues). The prey fish was then introduced to the empty side of the aquarium and was allowed a minute to orient itself before the tank separator was removed. The subsequent feeding event was filmed using a Go‐Pro (Hero 4) camera in real time, and a Sony RX100 IV to capture the predators’ strike in slow motion. Prey fish were removed after one minute if the predator failed to strike. If the predator made a nonlethal strike, the prey was immediately removed from the tank and euthanized using a clove oil anesthetic and ice‐water slurry. A successful capture by the predator was designated as the predator capturing and holding prey in its mouth for ≥3 s. After a successful feeding event, the predator had to fully digest the prey before another feeding trial could commence. This usually took two to four days and was assessed by visually inspecting for swelling in the stomach area of the predator and the behavior of the predator upon a researcher approaching the tank. A similar range of relative prey sizes was used across all predator morphotypes (based on prey body depth to predator gape). The majority (93%) of prey had a body depth over 45% of the predator gape, following ref. (Mihalitsis & Bellwood, 2017), to ensure predators performed close to their maximal abilities (Wainwright & Reilly, 1994).

Upon completion of feeding trials, the predator was euthanized using a clove oil anesthetic and an ice‐water slurry and the following morphological traits were measured: SL, total length (TL), and horizontal oral gape (sensu Mihalitsis & Bellwood, 2017). We also photographed the predator with its mouth closed and fully protruded, to quantify (using ImageJ) the predators’ ability to protrude its upper jaw. We note that the predator *Epibulus insidiator*, at maximal jaw protrusion, is unable to close its jaws and thus use its teeth; it was therefore classified as functionally edentulate. Photographs were also used to measure the eye size, which was later used as a scale in perpendicular strike videos (see below). Finally, the lateral head integument was removed, to reveal the structure of the predators’ adductor muscles (responsible for jaw closing). We recorded the extent of fusion between subdivisions of the adductor mandibulae (AM) (A1, A2, and A3) and their respective insertion sites. The AM complex was then removed and weighed to the nearest 0.001 g.

### 2.2 Image analyses

We extracted two datasets from our feeding videos. In the first, we recorded the capturing and processing behavior of piscivorous fishes. Traits quantified were as follows: body part struck, engulf versus grab, whether the predator used head‐shaking behavior postcapture, number of times the predator spat out and re‐ingested prey, and the direction of the preys’ body upon ingestion. Engulfing was defined as the majority (>90%) of the prey body being within the predators’ oral cavity upon a strike; grabbing was defined as the predator holding the prey between its oral jaw teeth on capture. In total, we recorded 90 successful feeding events.

In our second dataset, we analyzed only videos for which the predators’ strike was perpendicular to the camera, thus allowing the quantification of strike angle, strike distance, and the distance traveled by the predator postcapture. Distance traveled poststrike by the predator was only quantified if the strike did not appear to be influenced by potential interactions with the aquarium. Three snapshots were taken from each video recording: (1) just before strike initiation, (2) the moment at which prey was captured (for successful events) or predator strike was at maximal gape (for unsuccessful events), and (3) the furthest point reached following capture (see Figure S1). We then used the software Adobe Illustrator to join the snapshots together. We tilted and aligned the images, so that distances could be measured as straight horizontal lines (see Figure S1) using the software ImageJ. Images were scaled by the predator eye size. In total, we recorded 68 such feeding events.

### 2.3 Feeding performance and prey size in aquatic ecosystems: a meta‐analysis

We conducted a meta‐analysis of 2,209 published prey–predator size ratios (PPSR) in natural marine and freshwater ecosystems from published literature. We used the search engine Google Scholar and searched for terms relating to aquatic predation and predator and prey size (for published studies used, please see raw dataset provided). This analysis specifically examined prey body depth versus predator gape size; the key functionally relevant measurements for piscine predators (Mihalitsis & Bellwood, 2017). Data were only included if represented in terms of predator gape size versus prey body depth and in predators that were benthic rather than pelagic (sensu Mihalitsis & Bellwood, 2019a). This ensured that species in the meta‐analysis had similar habitat association to those examined in our experiments. We extracted the data using the software WebPlotDigitiser (Rohatgi, 2017) and classified the predators in the meta‐analysis based on the functional groups identified herein. If total length (TL) was not provided in the study, the recorded body size measurement was converted to TL using published morphometric relationships (Froese & Pauly, 2014).

### 2.4 Statistical analyses

All models and analyses were undertaken in the software R (R Core Team, 2017), using the packages *effects* (Fox, 2003; Fox & Weisberg, 2019), *emmeans* (Lenth, 2019), *car* (Fox & Weisberg, 2019), *ggplot* (Wickham, 2016), *nlme* (Pinheiro et al., 2014), *MuMIn* (Barton & Barton, 2019), *glmmTMB* (Brooks et al., 2017), and *stats* (R Core Team, 2017). Initially, we assessed whether there was a significant allometric effect on our morphological variables by plotting their body size (SL or weight) standardized values across body size (SL or weight, respectively). We found no evidence of significant allometry and therefore used standardized values. Morphological variables were also assessed in a phylogenetic context to evaluate the strength of phylogenetic influences. Phylogenetic tree construction was undertaken following Michonneau et al. (2016), and phylogenetic generalized least squares (PGLS) analyses follow Revell (2012) and Orme et al. (2012). To account for the effect of body size on morphological traits, adductor mandibulae (AM) mass was standardized through a PGLS regression of body mass versus AM mass, whereas remaining morphological traits were standardized through PGLS regressions with SL. The residuals of these relationships were then tested for differences between morphotypes. Lambda was estimated based on maximum likelihood, and evolution was assumed to follow a Brownian motion pattern (for phylogenetic tree used, see Figure S2). These results can be found in the Figure S3.

Strike angles (response variable) were also modeled using GLMMs following a Gaussian distribution and an identity link function, with individual id, nested within species, being the random effect. Strike angles (response) were modeled against morphotype (explanatory) and having species as a random effect.

For strike distance, we tested for a potential allometric effect with a linear model between body size (SL) and relative strike distance (strike distance/SL) and found no allometric effect (GLM; *p*‐value = .52). Strike distance was standardized to the predators’ body size (SL) to account for differences in predator body sizes. Analysis of strike distance was modeled using a GLMM, with a Gamma distribution, a log link function, and species being a random effect. Capture behavior among morphotypes was analyzed using a GLMM with a binomial distribution, a logit link function, and species being a random effect.

For all models, we used the Akaike information criterion (AIC) to determine the best model fit, following Zuur et al. (2013). Model validation (residual plots, Cooks’ distance etc.) followed ref. (Zuur et al., 2013); only suitable models were considered.

For the meta‐analysis, we modeled PPSRs (dependent variable) between the two functional groups identified herein and predator body size (independent variables) in a Bayesian framework. The model used a gamma distribution, a log link function, and default priors. Model estimation was performed using Markov chain Monte Carlo (MCMC) sampling. Three chains, with 5,000 iterations, a warm‐up of 2000, and a thinning factor of 5, were used. The model was run using the *rstanarm* (Goodrich et al., 2018) and *brms* (Bürkner, 2017) packages in R. Model residuals were simulated using the posterior predictive distribution and plotted using the *DHARMa* R package (Hartig, 2019), and model fit and assumptions were assessed using trace, autocorrelation, rhat, and effective sample size plots.

## 3 RESULTS

### 3.1 Morphology

**FIGURE 1:**
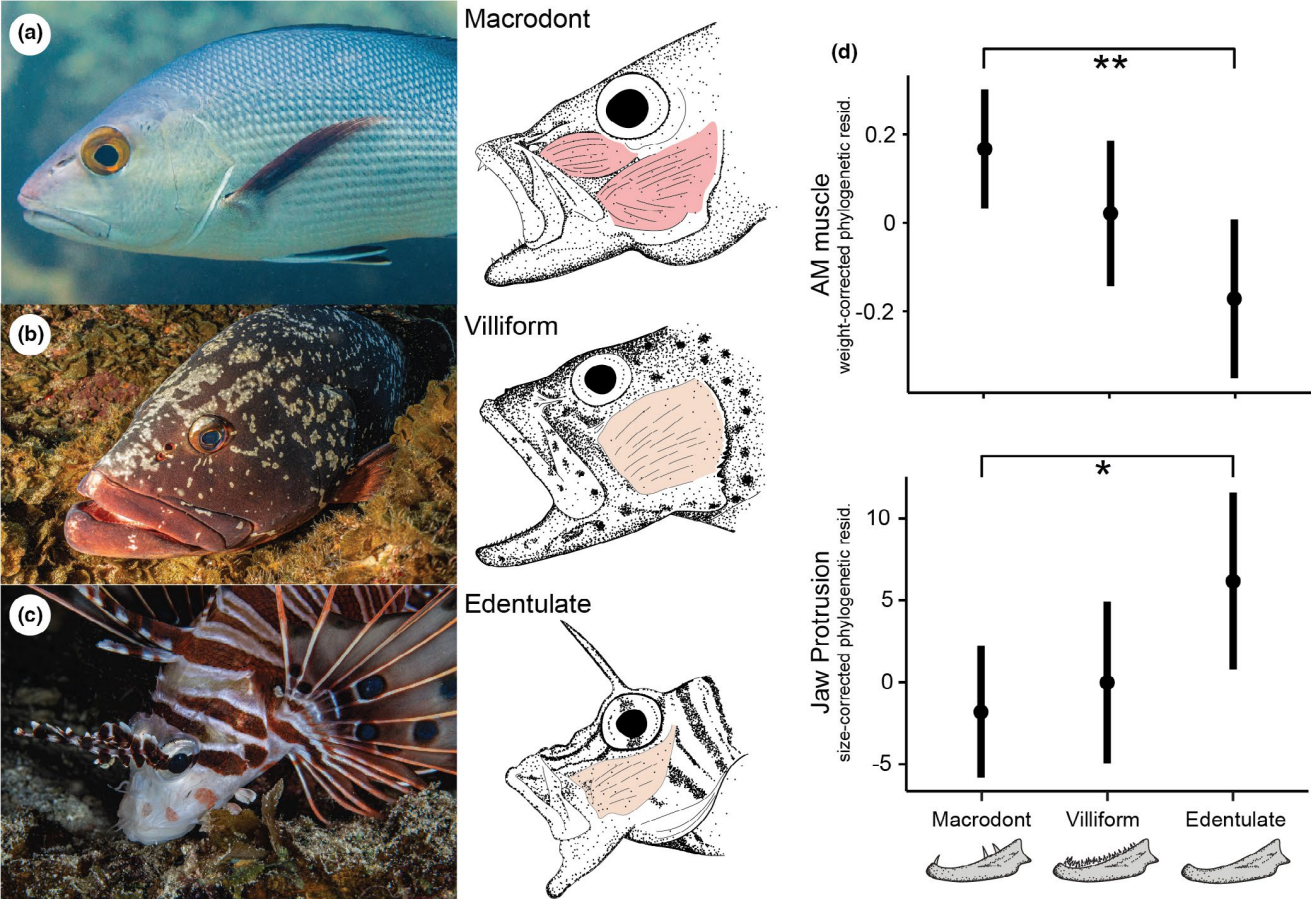
The three morphotypes investigated in our study. (a) macrodont, (b) villiform, (c) edentulate, following Mihalitsis and Bellwood (2019b). Illustrations show the myology of each morphotype, with macrodonts having distinct adductor mandibulae (AM) subdivisions, attaching to different parts of the maxillo‐mandibular ligament. Villiform and edentulate engulfers displayed fused AM subdivisions, with muscle fibers attaching along the length of the maxillo‐mandibular ligament. (d) macrodont morphotypes had a larger AM muscle mass than edentulate morphotypes (significance level indicated by asterisks). Edentulate morphotypes had higher jaw protrusion than macrodont morphotypes. Plots show mean predicted values for each group (± 95% confidence intervals). Photographs by Salvatore Di Lauro and Victor Huertas. Dentition illustrations modified after Mihalitsis and Bellwood (2019b)

We found significant differences in the feeding morphology of the three fish morphotypes (edentulate, villiform, and macrodont). Specifically, we found significant differences between the adductor mandibulae (AM) mass of macrodont and edentulate piscivores, with macrodonts having significantly larger AM (GLM; *p* < .01, Figure 1d). AM shape also varied among groups. Macrodonts displayed separated AM subdivisions (except for *Oxycheilinus sp*.) attaching at two primary locations on the maxillo‐mandibular ligament (Figure 1), whereas edentulate and villiform species displayed fused AM subdivisions (A1 and A2/A3), attaching along the entire length of the maxillo‐mandibular ligament (Figure 1). We also found significant differences in the jaw protrusion of macrodont and edentulate piscivores, with edentulate morphotypes having a significantly higher jaw protrusion ability (GLM; *p* < .05; Figure 1d). Essentially, from a morphological perspective, macrodonts had large, subdivided AM muscles, and low jaw protrusion ability, whereas edentulate morphotypes displayed small, fused AM muscles, and high jaw protrusion ability. Villiform morphotypes had an intermediate form between macrodont and edentulate morphotypes. These morphological differences strongly suggest that the three morphotypes will also exhibit distinct feeding performances. Experiments confirmed that this was the case.

### 3.2 Performance‐based experiments

**FIGURE 2:**
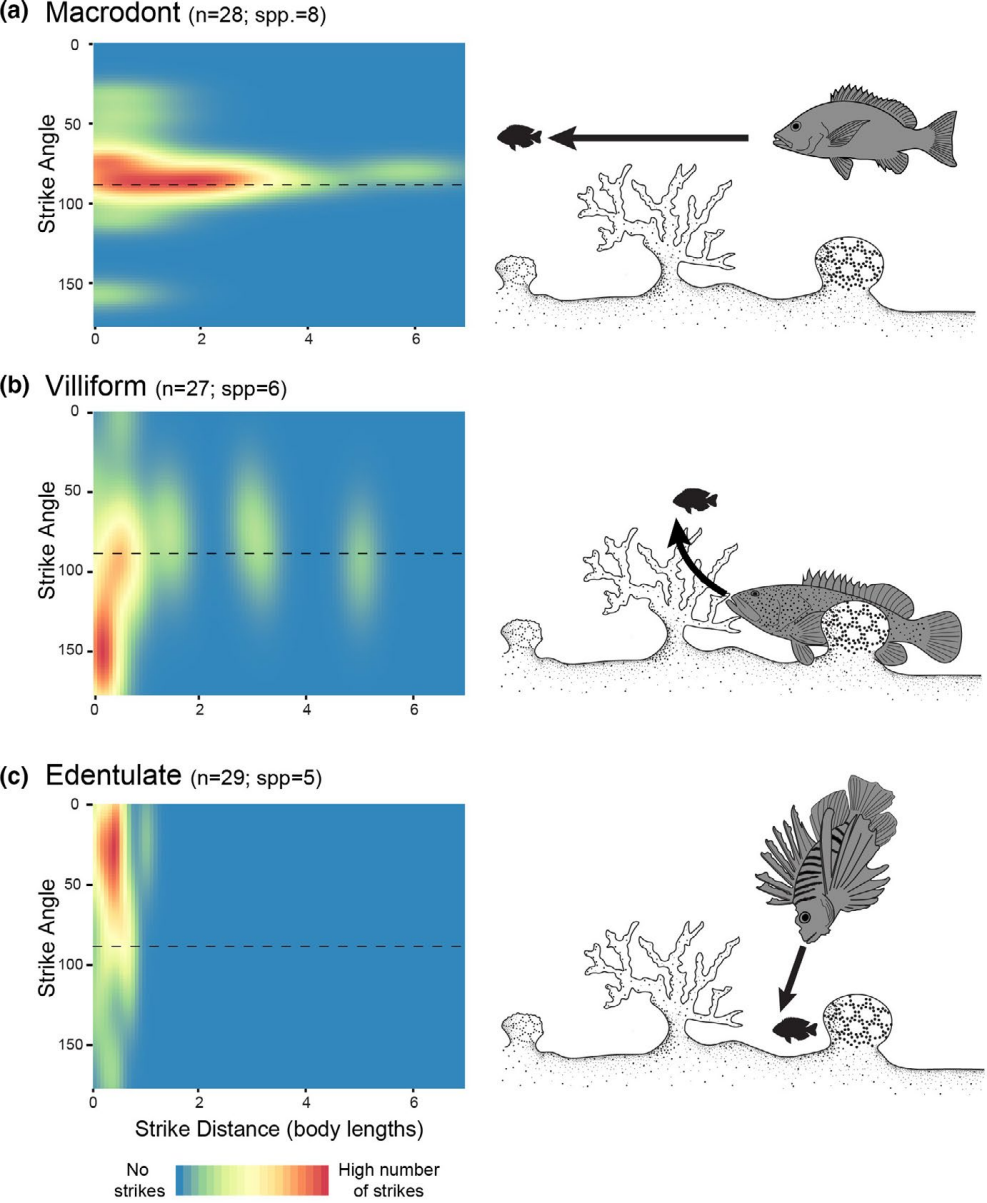
Heat maps showing the strike angle and strike distance of piscivorous coral reef fishes: (a) macrodont, (b) villiform, and (c) edentulate morphotypes. Macrodont piscivores are characterized by near horizontal, long‐distance strikes; villiform piscivores strike predominantly from below and close to their prey; edentulate piscivores strike primarily from short distances, from high angles above their prey. Illustrations highlight likely strike patterns on the reef

Both strike angle and strike distance differed significantly among piscivorous fish morphotypes (Figure 2). Villiforms were found to strike from significantly different angles compared to edentulate morphotypes (GLMM; *p* < .05, Table S3), with villiforms striking from high angles below the prey and edentulate morphotypes primarily striking from high angles above the prey (Figure 2). Basically, macrodonts strike from low (near horizontal) angles, whereas edentulate and villiform morphotypes strike from high angles. For strike distances, macrodont morphotype distances were significantly longer than either edentulate or villiform morphotypes (GLMM; *p* < .01; Figure 2, Table S3). Absolute standardized values of strike angle and strike distance showed a significant inverse relationship (GLM; *p* < .01, Figure S4). Overall, macrodont piscivores struck from low angles (approximately horizontal to the prey) from longer distances (>1 body length); villiform piscivores struck both from high angles under the prey with the strike directed upward, from a relatively short distance (usually <1 body length), and from low (horizontal) angles from a longer distance; edentulate piscivores struck from high angles above the prey, with the strike directed downward, and from a relatively short distance (<1 body length).

**FIGURE 3:**
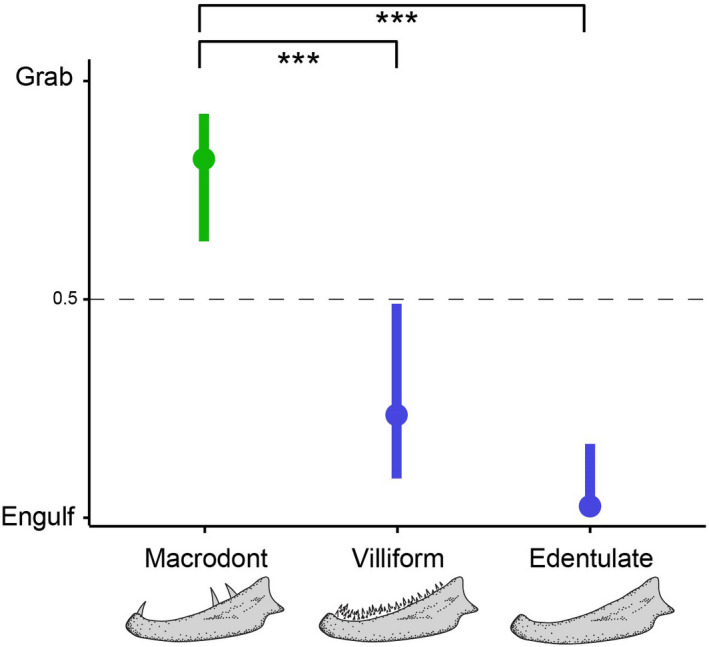
Capture behavior of piscivorous fishes. Macrodont piscivores predominantly capture their prey by grabbing (green color); villiform and edentulate piscivores capture their prey by engulfing (blue color). Plot shows mean predicted values for each group (± 95% confidence intervals). The horizontal dashed line represents the threshold between grabbing and engulfing. Significance level indicated by asterisks. Dentition illustrations modified after Mihalitsis & Bellwood (2019b)

Capture modes also differ between morphotypes with macrodonts differing significantly from both villiform and edentulate morphotypes (GLMM, *p* < .001; Figure 3). Macrodont piscivores primarily grabbed prey (83% of strikes; of these 84% were tailfirst and 16% body‐first) (Figure 3), whereas edentulate and villiform piscivores used engulfing as the primary capture mode (97% and 80% of strikes, respectively) (Figure 3). In essence, macrodont piscivores primarily feed by grabbing their prey tailfirst; edentulate and villiform piscivores primarily feed by engulfing their prey.

Of all grabbing strikes, only macrodont fishes followed with head‐shaking behavior, or hitting their prey against the base of the aquarium, resulting in prey laceration. After this behavior, they usually spat the prey out and re‐grabbed it headfirst before swallowing it. This behavior was also observed on the reef, in the macrodont *Oxycheilinus digramma* (Figure S5). Essentially, villiform dentitions were only observed to be used for capturing, whereas macrodont dentitions were used for both capture and postcapture processing.

Based on the morphological and behavioral results described above, we can identify two functional groups of piscivorous fishes: grabbers and engulfers. Grabbers encompass macrodont morphotypes, while engulfers encompass edentulate and villiform morphotypes.

### 3.3 Realized niche axis and ecosystem‐level implications: a meta‐analysis

**FIGURE 4:**
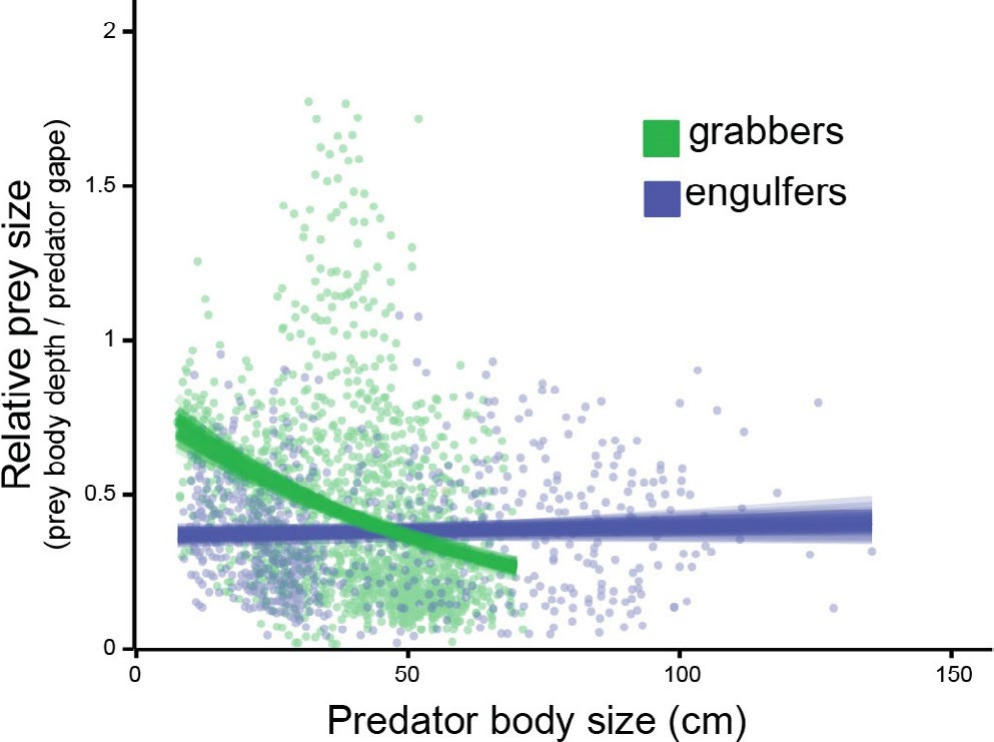
A meta‐analysis of trophic interactions in aquatic (marine and freshwater) ecosystems. Relative prey size (prey body depth/predator gape size) of piscivorous fishes versus predator body size for both grabbers (green) and engulfers (blue). Blue and green lines show randomly selected model fits selected from the posterior distribution for each functional group

We found clear evidence of resource partitioning in piscivorous fishes, along a relative prey size axis (Figure 4), with grabbing yielding larger relative prey (mean predator–prey size ratio: 0.42 with 0.40–0.43 95% CI), when compared to engulfing (mean predator–prey size ratio: 0.37 with 0.36–0.39 95% CI) (Figure 4) (see also Table S3 for model results). However, there appear to be ontogenetic changes for grabbers, with relative prey size decreasing as predator body size increases; for engulfers, this relationship does not appear to change with ontogeny (Figure 4, Table S3).

## 4 DISCUSSION

We found fundamental differences in the functional morphology, feeding behavior, and feeding niches of piscivorous fishes. These differences characterize two distinct functional groups: grabbers and engulfers (Table 1). We identify two distinct aspects of feeding: (1) based on how piscivores strike, capture, and process their prey, with clear evidence of resource partitioning, and (2) more extensive behavioral variation based on how predators behave prior to the strike. The functional groups identified herein complement previous terminologies and highlight the mechanistic basis of variation in the feeding behavior of piscivorous fishes.

**TABLE 1:**
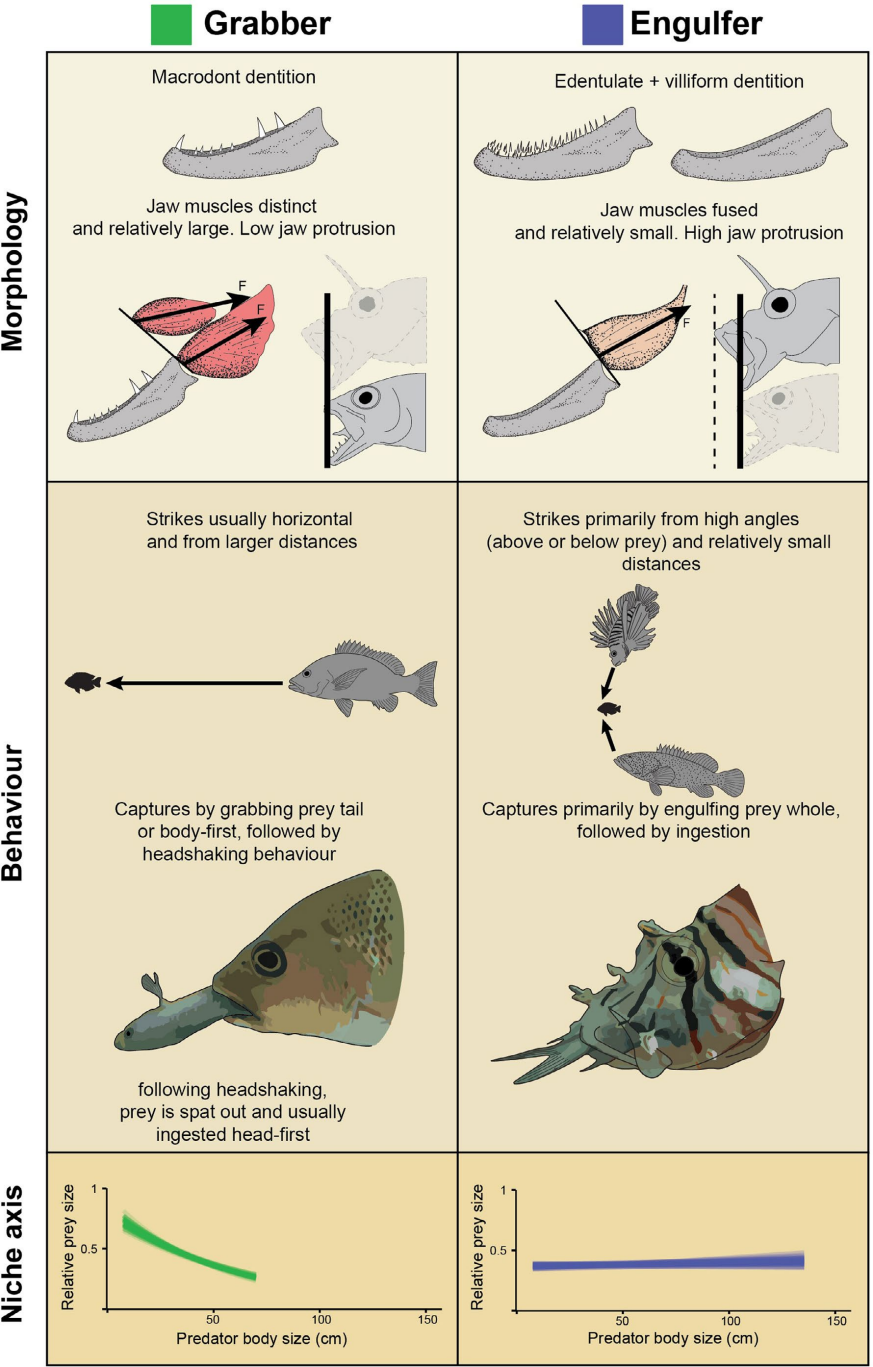
Summary of the morphology, behavior, and realized niche axis of grabbers and engulfers. Dentition illustrations modified after Mihalitsis & Bellwood (2019b)

### 4.1 Functional groups: grabbers

There is a clear axis of variation in piscivores. On one extreme, grabbers (primarily macrodont morphotypes) are characterized by longer strike distances from a horizontal position (Figure 2), with captures being primarily tailfirst. Previous work has found piscivores to be striking at the center of mass of prey fishes (Webb, 1986; Webb & Skadsen, 1980). The difference in capture location may be linked with the body shape of the prey. Moody et al. (1983) found the freshwater piscivore *Esox*, to be grabbing shallow‐bodied prey primarily mid‐body or tailfirst (49% and 37%, respectively), whereas deep‐bodied prey was captured primarily tailfirst (63%). Such results have been attributed to deep‐bodied bluegills (*Lepomis macrochirus*) being more difficult to capture, as opposed to shallow‐bodied fathead minnows (*Pimephales promelas*) (Gillen et al., 1981; Wahl & Stein, 1988). These differences in capture location on the prey's body (and ultimately the strike outcome) may also be reflected in the wild, where prey availability consists of both deep‐bodied and shallow‐bodied functional groups of prey (sensu Mihalitsis et al., 2021).

Furthermore, the location of capture along the preys' body may be related to the predators' jaw morphology. Jaw elongation in aquatic predators creates a velocity advantage at the tip of the jaw. Such increased velocity may, however, decrease the accuracy of the strike, thus resulting in the predator striking at the body part suggested to move least in fast escape response (Webb & Skadsen, 1980; Weihs, 1973). Indeed, most studies that mention prey being captured at the center of mass appear to be predominantly conducted with piscivores that have elongated jaw morphologies (e.g., *Lepisosteus*) (Porter & Motta, 2004; Webb & Skadsen, 1980) and feed by positioning themselves next to the prey and conducting a high‐speed lateral head movement (Porter & Motta, 2004). This was also found in one of the most extreme cases of jaw elongation and feeding through lateral head movement in sailfish and marlins (Domenici et al., 2014; Hansen et al., 2020) (Video S1 of their study showing sailfish capturing prey at center of mass). Interestingly, jaw length has been found throughout multiple major taxa to be a primary axis of morphological variation (Arbour et al., 2020; Martinez et al., 2018, 2021; Price et al., 2019). Such patterns of jaw elongation and dentition have also been found in other vertebrate taxa, such as crocodylomorphs (Stubbs et al., 2013).

Tailfirst captures could also be a product of the prey noticing the predator and initiating an escape response before capture, given that grabbers were found striking from relatively longer distances. It is reported that schooling fishes have a “slower” response to predator strikes when compared to solitary fishes (Domenici & Batty, 1997). Given that grabbers may strike from longer distances and that schooling fishes (on reefs) are found further away from the benthos (Hobson, 1965) suggests that grabbers may be more successful at feeding on schooling fishes in the water column.

Essentially, grabbers, because of their capacity to strike from a longer distance, may have an advantage when targeting schooling fishes as they may have a performance‐based competitive advantage over engulfers that strike from close distances. This scenario is consistent with field evidence. On the reef, the grabber *Plectropomus leopardus* has been found to be feeding predominantly on pomacentrids and other social fishes in the water column (Matley et al., 2018; St. John et al., 2001). Benthic taxa such as gobies and blennies, which are also highly abundant on coral reefs, were almost absent from their diet. These observations, along with the difficulties associated with a body ram strike toward the benthos (Mihalitsis et al., 2021), strongly suggest that macrodont grabbers are better suited for feeding on prey swimming in the water column.

### 4.2 Functional groups: engulfers

At the other extreme, engulfers (edentulate and villiform morphotypes) were found to strike from short distances, at high angles from above or below, and primarily engulf their prey. This relationship between strike distance and angle appears to be strongly linked (Figure S4). This suggests that a grabbing strike may require more space relative to an engulfing strike. Indeed, grabbing strikes are often observed in open pelagic waters, whereas engulfing strikes are primarily observed in benthic‐associated predators.

Morphological specializations associated with this feeding mode, such as jaw protrusion, have been found to enhance the suction ability of fishes (Holzman et al., 2008; Staab et al., 2012). The combination of high jaw protrusion and enhanced suction abilities appears to have evolved for feeding on elusive prey, especially those associated with the benthos (Bellwood et al., 2015; Higham et al., 2006). The inertia associated with long‐distance high‐velocity strikes (Tran et al., 2010; Wainwright et al., 2001) may result in the predator injuring its jaws and/or teeth against the substratum if a body ram strike is used on a benthic prey fish (e.g., gobies). Furthermore, prey that are strongly associated with the benthos may constrain the potential success of grabbing predators because of the need to identify the precise location for a grabbing bite. When using jaw protrusion and suction, there are fixed biomechanical limitations on jaw excursion, that is, in the extent to which the jaw can extend. Furthermore, the predators’ body will act as an anchor in stopping the predator from moving postcapture, following jaw protrusion. Thus, strike distance can be carefully controlled. This feeding behavior closely matches field observations, that is, strikes from close‐range using jaw protrusion to engulf prey (see the engulfer *Pterois volitans,* Figure S5) (see also Collins & Motta, 2017; Green et al., 2019). Essentially, these traits (jaw protrusion and enhanced suction) may provide engulfers with distinct advantages in reef environments, as they appear to be exceptionally well suited for accessing prey that are closely associated with the substratum.

### 4.3 Linking functional groups to previous terminology

**FIGURE 5:**
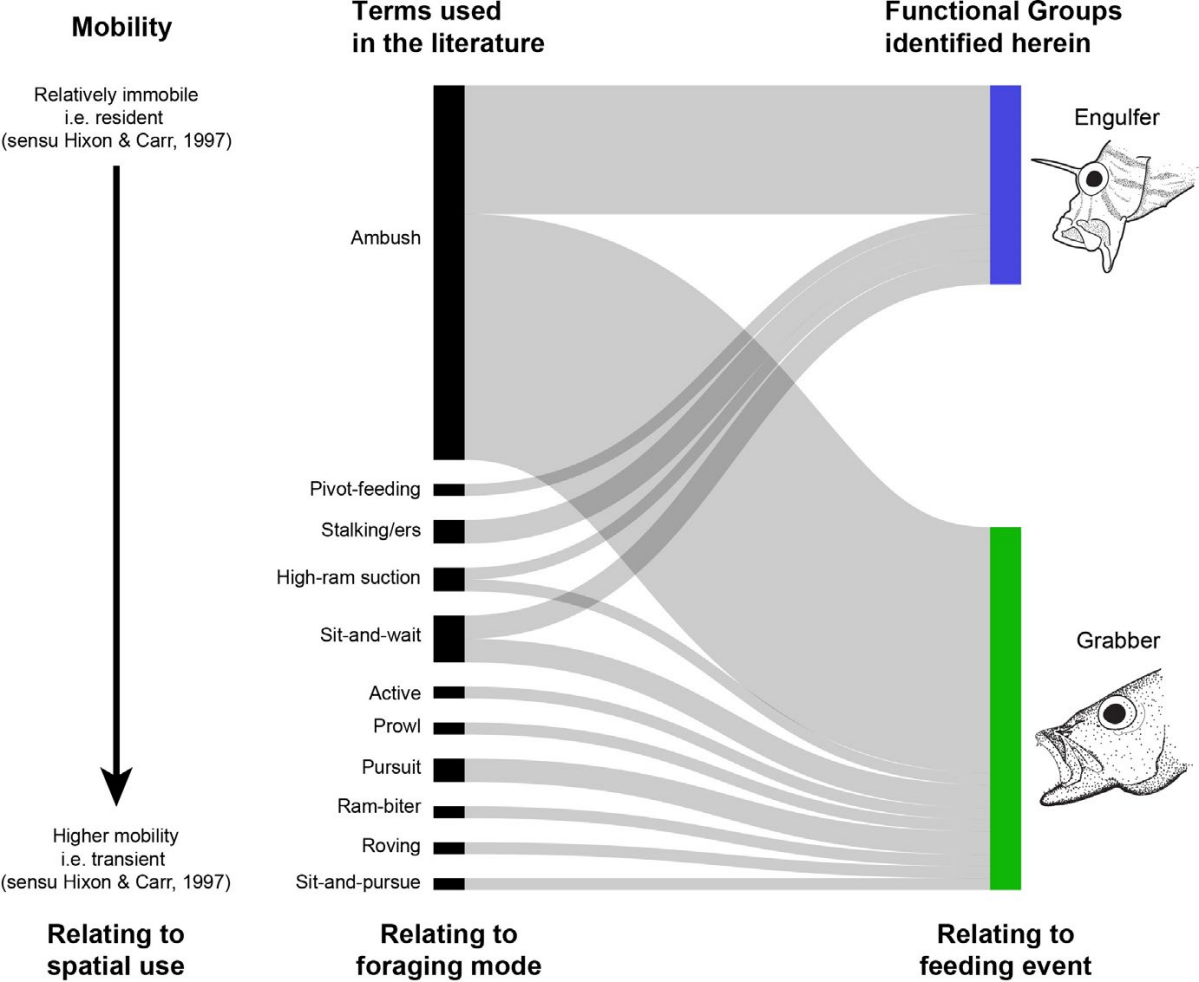
Classification of predatory/piscivorous fishes. The figure relates classification terms used in the literature to the functional groups identified herein. The “Mobility” column highlights an axis of low‐to‐high activity, reflecting the resident versus transient distinction of Hixon and Carr (1997). While previous terms used in the literature refer primarily to the predators' foraging mode (i.e., activity leading up to the feeding event), or an aspect of its hunting behavior (e.g., pursuit), our functional groups relate directly to the feeding event (timeframe of few seconds) and link the functional feeding morphology of the predator to its striking, capturing, and processing behavior

Classifications of piscivorous fish groups are widespread in the literature and incorporate terms such as ambush versus pursuit, transient versus resident, ram versus suction etc. When reviewing the literature, we found 11 different terms describing different types of feeding behaviors in predatory fishes (Figure 5). Furthermore, we found the term “ambush” to be used for multiple types of piscivorous fishes with different feeding morphologies. For example, *Pterois volitans, Epinephelus maculatus*, and *Plectropomus leopardus* are all termed ambush predators. However, these species display highly differentiated functional feeding traits, having fundamentally different dentitions (respectively edentulate, villiform, and macrodont) (sensu Mihalitsis & Bellwood, 2019). We also found the same species to be classified with different terms in different studies. For example, we found the grabber *Pseudochromis fuscus* to be classified as both an ambush and pursuit predator (see Table S1). Such inconsistencies likely arise by classifying predators based on different aspects related to either morphology (i.e., biters, suction‐feeders) or behavior. Within behavior, classifications have been further divided based on different aspects, such as striking behavior (i.e., ambush, pursuit) or spatial behavior (i.e., resident, transient).

The classifications identified in our current study (Figure 5) are based on principles denoting functional morphology (or ecomorphology) (Wainwright & Bellwood, 2002; Wainwright & Reilly, 1994). Following principles of this field, morphological attributes (i.e., traits) are tested in an experimental, performance‐based context (testing their maximal abilities), to inform how organisms are able to use these tools (i.e., morphology) to carry out different tasks (i.e., behavior). Performance experiments help to distinguish between spurious correlations and morphological attributes used by the organism in these tasks. As a result, such studies have been able to link functional morphology, to performance, to behavior, and, finally, to realized niches (Fulton et al., 2017; Huertas & Bellwood, 2017; Wainwright, 1987, 1988).

In this context, our groups relate to the final moment of the strike. However, there is a much broader array of classifications which relate to different aspects of the feeding strategies of these fishes and how this leads to the capture of prey (Figure 5). Such classifications may extend to aspects relating to the entire lifestyle of the predator (i.e., ambush), the approaching technique it utilizes (i.e., stalking), or the strike initiation (i.e., pursuit). The functional groups identified herein relate to the few seconds/minutes between strike initiation and prey ingestion and encompass morphology and behaviors related to striking, capturing, and processing.

### 4.4 Ecological implications

Most studies, when quantifying predator–prey size relationships, tend to quantify predator versus prey relationships as a standard length versus standard length relationship (Gaeta et al., 2018; Scharf et al., 2000). However, body depth is arguably the major axis of variation in fishes (Claverie & Wainwright, 2014; Friedman et al., 2019), as well as being the limiting factor in gape limitation for piscivorous fishes (Mihalitsis & Bellwood, 2017; Nilsson & Brönmark, 2000; Wainwright & Richard, 1995). While SL versus SL relationships may be beneficial for studies focusing on population structure, they may mask the mechanistic basis of functional relationships between predators and prey (e.g., Mihalitsis et al., 2021). Our results suggest that shifting this relationship to a predator gape size versus prey body depth relationship and incorporating their functional signature may provide a mechanistic, causal, link between the functional morphology or behavior and functional role of piscivorous fishes in ecosystems (Figure 4) (e.g., Dörner & Wagner, 2003). For example, our results suggest that piscivory (i.e., prey removal) may be separated into the piscivores that predominantly remove relatively large prey versus small prey (Figure 4) and that the “who” removes large versus small prey changes with increasing body size.

Differences in the composition of piscivores, therefore, may influence the size structure of prey fish communities (cf. Mihalitsis et al., 2021). Juveniles of a certain species (and therefore smaller body size) focus on growth, while larger individuals focus more on reproduction (Barneche et al., 2018; Morais & Bellwood, 2020; Roff, 1983). By feeding on “growth‐focused” individuals versus “reproduction‐focused” individuals, piscivores may disproportionately influence the productivity potential of a fish community. By quantifying predator body size (that can then be transformed to gape size) and incorporating piscivore functional groups when surveying the piscivorous fish community on a coral reef may provide critical insights into the potential predation pressure and its size specificity.

### 4.5 Evolutionary implications

**FIGURE 6:**
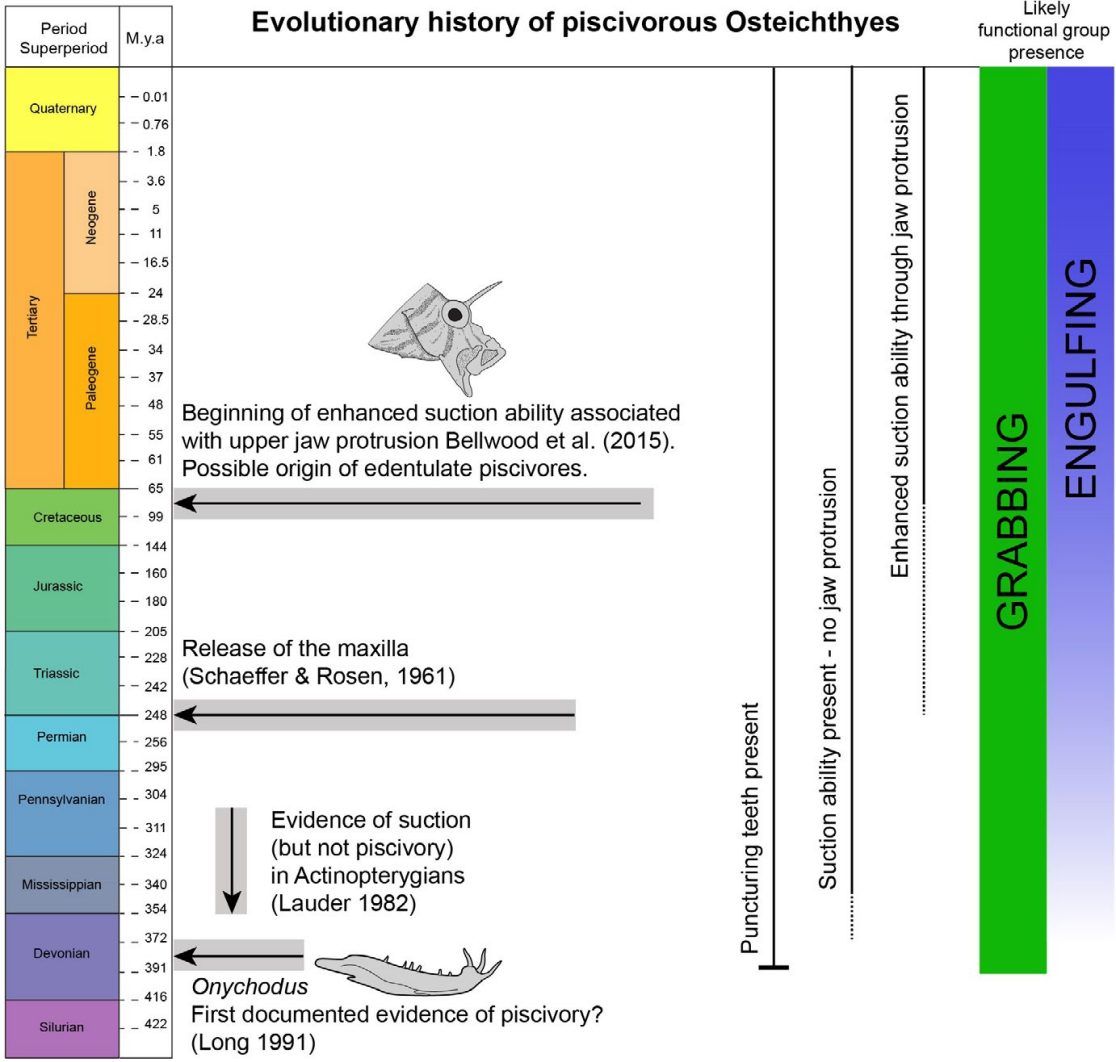
Evolutionary history of piscivorous Osteichthyes. Evidence of grabbing within the Osteichthyes has been found dating back to the Devonian, with the sarcopterygian *Onychodus*. Early actinopterygians have been shown to be able to use suction (Lauder, 1982); yet, how much this contributes to prey capture relative to body and jaw ram (jaw protrusion) remains unknown, and there is currently no direct link to piscivory. Increased jaw protrusion (Bellwood et al., 2015), leading to enhanced suction abilities (Staab et al., 2012), is only seen more recently in the Late Cretaceous and is a common feature of many extant piscivores

Macrodont fishes appear to be the first recorded piscivorous morphotype in the evolution of bony fishes (Osteichthyes) (Figure 6). To our knowledge, the first evidence of macrodont dentition directly associated with piscivory is in the Late Devonian sarcopterygian *Onychodus* (Andrews et al., 2005; Long, 1991). Furthermore, Long (1991) described a fossil of an *Onychodus* having captured and ingested a placoderm (Placodermi). In keeping with our results, Long (1991) suggests that the predator captured the prey fish tailfirst. This evidence, along with our results, suggests that “grabbing” as a means of capturing elusive prey, already existed in the Devonian (419.2–358.9 Mya). Grabbing (and by association body ram striking) as a means of prey capture may therefore have arisen before engulfing (and by association jaw ram striking), which requires further morphological modifications (Figure 6).

Within actinopterygians, however, it is still unclear which of the two feeding behaviors arose first. Lauder (1985) suggested that suction feeding was a basal trait in the Osteichthyes, and Lauder (1980) demonstrated the ability of the primitive actinopterygian *Amia calva* to use suction as a means of feeding. Indeed, *A.calva* shows reduced, curved, and compact dentition, more aligned with villiform dentition, primarily used for holding as opposed to puncturing flesh during a grabbing strike (sensu Mihalitsis & Bellwood, 2019b). Furthermore, it is not clear to what extent the origin of suction was associated with piscivory, and the contribution of suction relative to other mechanisms (e.g., jaw and body ram) in the early actinopterygian fishes has, to our knowledge, yet to be quantified. The distinction of whether fishes are able to use suction versus how much suction contributes to prey capture is an important distinction, as noted by Longo et al. (2016).

Engulfing involves, and probably requires, some degree of jaw protrusion and suction, and it therefore requires specific modifications of the cranial morphology. Jaw protrusion in actinopterygians was triggered by the release of the maxilla from the preopercular and infraorbital bones, at some point during the Late Permian (256–248 Mya) (Schaeffer & Rosen, 1961). Subsequent expansion and specialization of this trait has been identified as a major modification facilitating the capture of elusive prey by fishes (Bellwood et al., 2015). Engulfing via jaw protrusion thus appears to be a relatively recent feeding mode, when compared to grabbing (Figure 6). Although the fish investigated herein are coral reef fishes, the functional groups identified in our study are likely to apply to fishes from any aquatic environment (Arbour et al., 2020; Camp et al., 2015; Keppeler et al., 2020; Weller et al., 2020).

### 4.6 Future Implications

Piscivorous fishes are primary targets in many coral reef fisheries (e.g., Cinner et al., 2009; Dulvy et al., 2004; Graham et al., 2005; Madin et al., 2016; Valdivia et al., 2017). The implications of this removal on ecological functions remain unknown. Most coral reef fisheries catch‐data are analyzed from a taxonomic, trophic guild, or trait‐based approach (Cinner et al., 2009; Russ & Alcala, 1989). Such studies have been useful in shifting the focus from a biodiversity‐based perspective to a more mechanistic or functional perspective (Bellwood et al., 2004). However, to date, functional evaluations of coral reefs have focused predominantly on herbivores (Bellwood et al., 2012; Robinson et al., 2020). Our work suggests that future studies may also need to incorporate different functional groups of piscivorous fishes. Fisheries may be removing different functional groups of piscivorous fishes disproportionately, changing both the composition of piscivorous fishes and their functional role in reef ecosystems. The ecological implications of the removal of functional groups within piscivorous coral reef fishes are unknown, but given the overwhelming importance of piscivory in energetic and nutrient flows, their role may be an important one.

Furthermore, our observations suggest that fishes along the grabbers to engulfers axis may also differ in their dependency on structural complexity. It is well documented that coral reefs in the Anthropocene are losing topographic complexity and that they are turning into more flattened, less structurally complex environments (Hughes et al., 2017; Zawada et al., 2019). Getting close to potential prey for a short‐distance strike may therefore become more challenging in the future. Piscivorous fishes may thus be subject to both direct and indirect human disturbance.

Overall, we show that piscivores are not a uniform group, but a spectrum of different functions and modes. Specifically, there are two different functional groups of benthic piscivorous fishes, based on their functional morphology, striking, capturing, and processing behavior. We identify a major axis of variation in the feeding behavior of piscivorous fishes, grabbing versus engulfing. These results suggest that a separation of piscivorous fishes into functional groups may be valuable in future studies, as different groups are likely to have significant implications for both functional and community ecology.

## CONFLICT OF INTEREST

The authors declare no conflict of interest.

## AUTHOR CONTRIBUTION

**Michalis Mihalitsis:** Conceptualization (equal); Data curation (lead); Formal analysis (lead); Investigation (lead); Methodology (equal); Visualization (lead); Writing‐original draft (lead); Writing‐review & editing (equal). **David R. Bellwood:** Conceptualization (equal); Data curation (supporting); Formal analysis (supporting); Funding acquisition (lead); Investigation (equal); Methodology (supporting); Resources (equal); Supervision (lead); Visualization (supporting); Writing‐original draft (equal); Writing‐review & editing (equal).

## Supporting information

Appendix S1

## Data Availability

Data deposited in the Dryad Digital Repository (https://doi.org/10.5061/dryad.b5mkkwhdp).
