# Supplementary material for: Functional groups in piscivorous fishes
Source: Ecol Evol. 2021 Sep 2;11(18):12765–78. doi: 10.1002/ece3.8020 (PMC8462170; doi:10.1002/ece3.8020)
Supplement: Supplementary file 1 — Appendix S1 [file ECE3-11-12765-s001.docx]

**Supplemental Information**

*Manuscript: Functional groups in piscivorous fishes*

*Michalis Mihalitsis^1,2^, David R. Bellwood^1,2^*

| **Term used in literature** | **Species** | **Morphotype** | **Functional Group** | **Reference** |
| --- | --- | --- | --- | --- |
| Ambush | *Cephalopholis cruentata* | V | GR | Green et al. 2019 |
| Ambush | *Plectropomus leopardus* | M | GR | Wen et al. 2013, St. John 1995 |
| Ambush | *Pseudochromis fuscus* | M | GR | Wen et al. 2013, Feeney et al. 2012 |
| Ambush | *Epinephelus morio* | V | EN | Freitas et al. 2017 |
| Ambush | *Mycteroperca bonaci* | V | GR | Freitas et al. 2017 |
| Ambush | *Synanceia verrucosa* | V | EN | Grobecker 1983 |
| Ambush | *Cephalopholis cruentata* | V | GR | Almany 2004 |
| Ambush | *Cephalopholis fulva* | V | GR | Almany 2004 |
| Ambush | *Serranus tigrinus* | V | GR | Almany 2004 |
| Ambush | *Rypticus bistrispinus* | E | EN | Almany 2004 |
| Ambush | *Paracirrhites arcatus* | M | GR | DeMartini 1996, Hobson 1974, |
| Ambush | *Trachinocephalus uranoscopus* | V | GR | Kwik 2011 |
| Ambush | *Synodus intermedius* | V | GR | Rojas-Velez et al. 2019 |
| Ambush | *Sphyraena barracuda* | M | GR | Rojas-Velez et al. 2019 |
| Ambush | *Paracirrhites forsteri* | M | GR | Leray et al. 2012 |
| Ambush | *Paracirrhites hemistictus* | M | GR | Leray et al. 2012 |
| Ambush | *Epinephelus marginatus* | V | EN | Gerhardinger et al. 2006 |
| Ambush | *Synodus englemani* | V | GR | Sweatman 1984 |
| Ambush | *Synodus dermatogenys* | V | GR | McCormick et al. 2018 |
| Ambush | *Cephalopholis microprion* | V | GR | McCormick et al. 2018 |
| Ambush | *Pterois volitans* | E | EN | Morris, Akins 2009 |
| Ambush | *Cephalopholis boenak* | V | GR | Palacios et al. 2018 |
| Ambush | *Epinephelus maculatus* | V | EN | Palacios et al. 2018 |
| Ambush | *Synodus variegatus* | V | GR | Holmes McCormick 2006 |
| Ambush | *Epinephelus itajara* | V | EN | Collins Motta 2017 |
| Ambush | *Lates calcarifer* | E | EN | Norin and Clark 2017 |
| Ambush | *Trichiurus lepturus* | M | GR | Bemis et al. 2019 |
| Ambush | *Dendrochirus zebra* | E | EN | Moyer and Zaiser 1981 |
| Ambush | *Aulostomus maculatus* | E | EN | Aronson 1983 |
| Ambush | *Antennarius hispidus* | E | EN | Longo et al. 2016 |
| Ambush | *Cephalopholis hemistiktos* | V | GR | Shpigel and Fishelson 1989 |
| Ambush | *Cephalopholis moiniata* | V | GR | Shpigel and Fishelson 1989 |
| Pursuit | *Pseudochromis fuscus* | M | GR | McCormick et al. 2018 |
| Pursuit | *Thalassoma lunare* | M | GR | McCormick et al. 2018 |
| Sit-and-wait | *Mycteroperca bonaci* | V | GR | Preisser et al. 2007, Parrish 1993, Catano et al. 2017 |
| Sit-and-wait | *Cephalopholis boenak* | V | GR | Palacios et al. 2018 |
| Sit-and-wait | *Epinephelus maculatus* | V | EN | Palacios et al. 2018 |
| Sit-and-wait | *Pterois volitans* | E | EN | Cure et al. 2012 |
| Sit-and-pursue | *Sphyraena barracuda* | M | GR | Preisser et al. 2007, Catano et al. 2017 |
| Stalking/ers | *Pterois volitans* | E | EN | Green et al. 2019, Cure et al. 2012 |
| Stalking/ers | *Dendrochirus zebra* | E | EN | Moyer and Zaiser 1981 |
| Active | *Pseudochromis fuscus* | M | GR | Preisser et al. 2007, Palacios et al. 2018 |
| Roving | *Lutjanus apodus* | M | GR | Green et al. 2019 |
| Prowl | *Plectropomus leopardus* | M | GR | St. John 1995 |
| Pivot-feeding | *Aulostomus maculatus* | E | EN | Longo et al. 2016 |
| High-ram suction | *Epinephelus ongus* | V | EN | Longo et al. 2016 |
| High-ram suction | *Caranx sexfasciatus* | E | GR | Longo et al. 2016 |
| Ram-biter | *Sphyraena barracuda* | M | GR | Longo et al. 2016 |

**Supplemental Table 1.** Data used for Figure 5 in the main text.


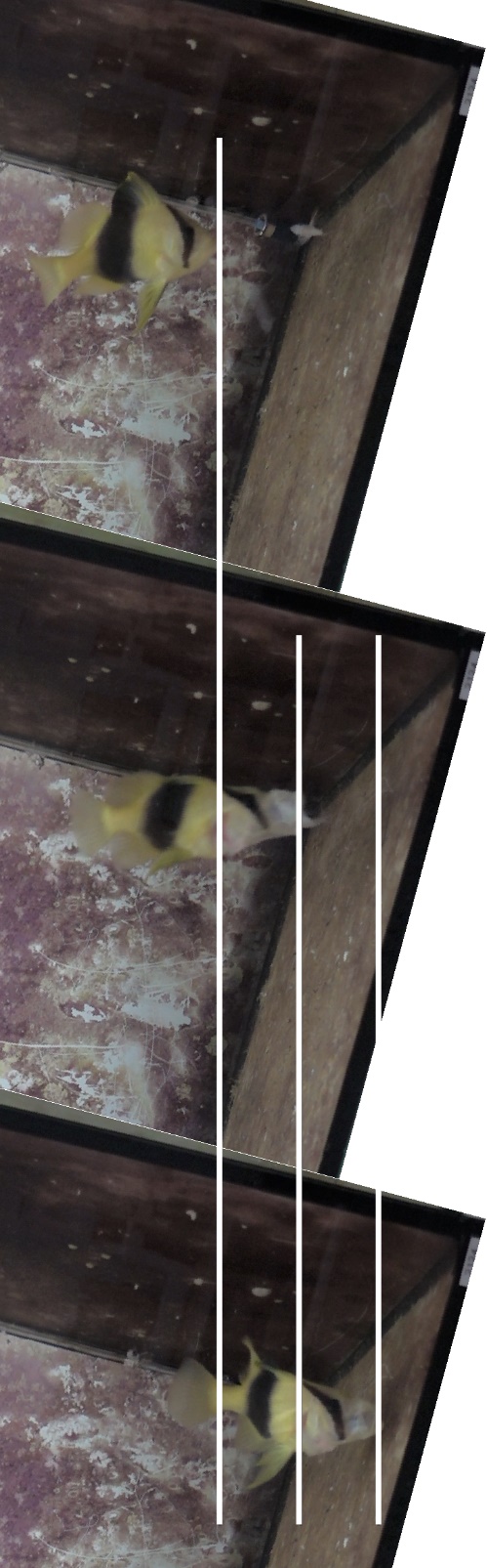


**Supplemental Figure 1**: Frames from videos used in our analyses. Vertical lines represent points from which strike distances, and distance travelled post captured were measured. Frames represent strike initiation, moment of capture, and frame at which strike is completed.


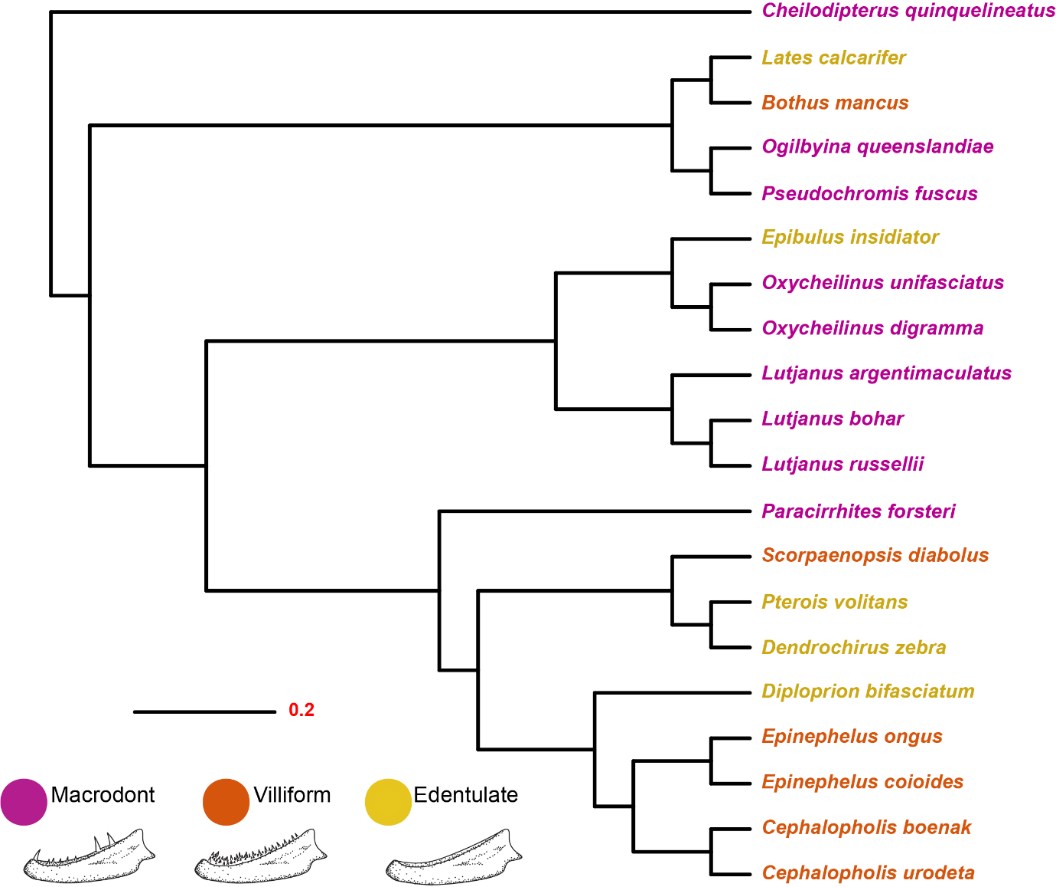


**Supplemental Figure 2**: Phylogenetic tree used in analyses.

**Supplemental Table 2**: Statistical models used to compare morphological variables between morphotypes, on body size and phylogenetic-corrected residuals.

| *glm(Dependent ~ Morphotype)* |  |  |  |  |  |
| --- | --- | --- | --- | --- | --- |
| **Variable** | **Level** | **Estimate** | **St. Error** | **t-value** | **p-value** |
| Protrusion | (Intercept) | 6.176 | 2.752 | 2.244 | 0.0384 |
|  | Macrodont | -7.978 | 3.432 | -2.324 | **0.032** |
|  | Villiform | -6.186 | 3.726 | -1.66 | 0.115 |
| Adductor Mandibulae | (Intercept) | -0.172 | 0.091 | -1.871 | 0.078 |
|  | Macrodont | 0.339 | 0.114 | 2.96 | **<0.01** |
|  | Villiform | 0.193 | 0.124 | 1.556 | 0.138 |
| Gape size | (Intercept) | -0.524 | 4.584 | -0.114 | 0.91 |
|  | Macrodont | -0.9 | 5.717 | -0.158 | 0.877 |
|  | Villiform | 8.86 | 6.207 | 1.427 | 0.172 |

**Supplemental Table 3**: Statistical models used in behavioural analyses and metanalysis.

| **Strike behaviour** | **glmm(Angle ~ Morphotype + (1\|Species), REML = T)** |  | **Estimate** | **Std. Error** | **z value** | **p-value** |
| --- | --- | --- | --- | --- | --- | --- |
|  |  | (Intercept) | 65.86 | 16.47 | 3.999 | <0.001 |
|  |  | Macrodont | 13.53 | 21.43 | 0.632 | 0.5277 |
|  |  | Villiform | 50.65 | 22.65 | 2.236 | **0.0254** |
|  | **glmm(Strike Distance ~ Morphotype + (1\|Species), family=Gamma(link='log'),REML=T)** |  | **Estimate** | **Std. Error** | **z value** | **p-value** |
|  |  | (Intercept) | -0.6601 | 0.2826 | -2.336 | 0.0195 |
|  |  | Macrodont | 1.127 | 0.3696 | 3.049 | **0.0023** |
|  |  | Villiform | 0.3817 | 0.3915 | 0.975 | 0.3295 |
|  | **glm(Angle ~ poly(Strike distance,2), family=Gamma(link='log'))** |  | **Estimate** | **Std. Error** | **t value** | **p-value** |
|  |  | (Intercept) | 0.1191 | 0.138 | 0.863 | 0.391 |
|  |  | Strike distance | -0.486 | 0.089 | -5.465 | **<0.001** |
| **Capture behaviour** | **glmm(Capture ~ Morphotype+(1\|Species),family = binomial(link='logit'),REML=T)** |  | **Estimate** | **Std. Error** | **z value** | **p-value** |
|  |  | (Intercept) | 0.0236 | 0.0252 | -3.51 | <0.001 |
|  |  | Macrodont | 175 | 209 | 4.32 | **<0.001** |
|  |  | Villiform | 7.44 | 9.07 | 1.65 | 0.09 |
| **Post-capture** | **glmm(Distance after strike ~ Morphotype + (1\|id/Species), family = 'Gamma'(link = 'log'), REML = T)** |  | **Estimate** | **Std. Error** | **z value** | **Pr(>\|z\|)** |
|  |  | (Intercept) | 2.3394 | 0.2973 | 7.87 | <0.001 |
|  |  | Macrodont | 1.2822 | 0.3965 | 3.234 | **0.00122** |
|  |  | Villiform | 1.0031 | 0.4205 | 2.386 | **0.01705** |
| **Metanalysis** | **stan_glm(prop_prey_size ~ FG2*pred_length, data=psdata,  family=Gamma(link ='log'),refresh=0,  chains=3,iter=5000,warmup=2000,thin=5,  adapt_delta=0.99)** |  | **estimate** | **std.error** | **conf.low** | **conf.high** |
|  |  | (Intercept) | -1.02 | 0.04 | -1.09 | -0.934 |
|  |  | Functional group grab | 0.809 | 0.066 | 0.674 | 0.931 |
|  |  | pred_length | 0.0000771 | 0.0000791 | -0.0000796 | 0.000227 |
|  |  | Functional group grab:pred_length | -0.00169 | 0.000148 | -0.00199 | -0.00141 |
|  |  | shape | 2.76 | 0.0786 | 2.6 | 2.9 |
|  |  | mean_PPD | 0.412 | 0.00755 | 0.398 | 0.427 |
|  |  | log-posterior | 268 | 1.59 | 265 | 270 |
|  |  |  |  |  |  |  |


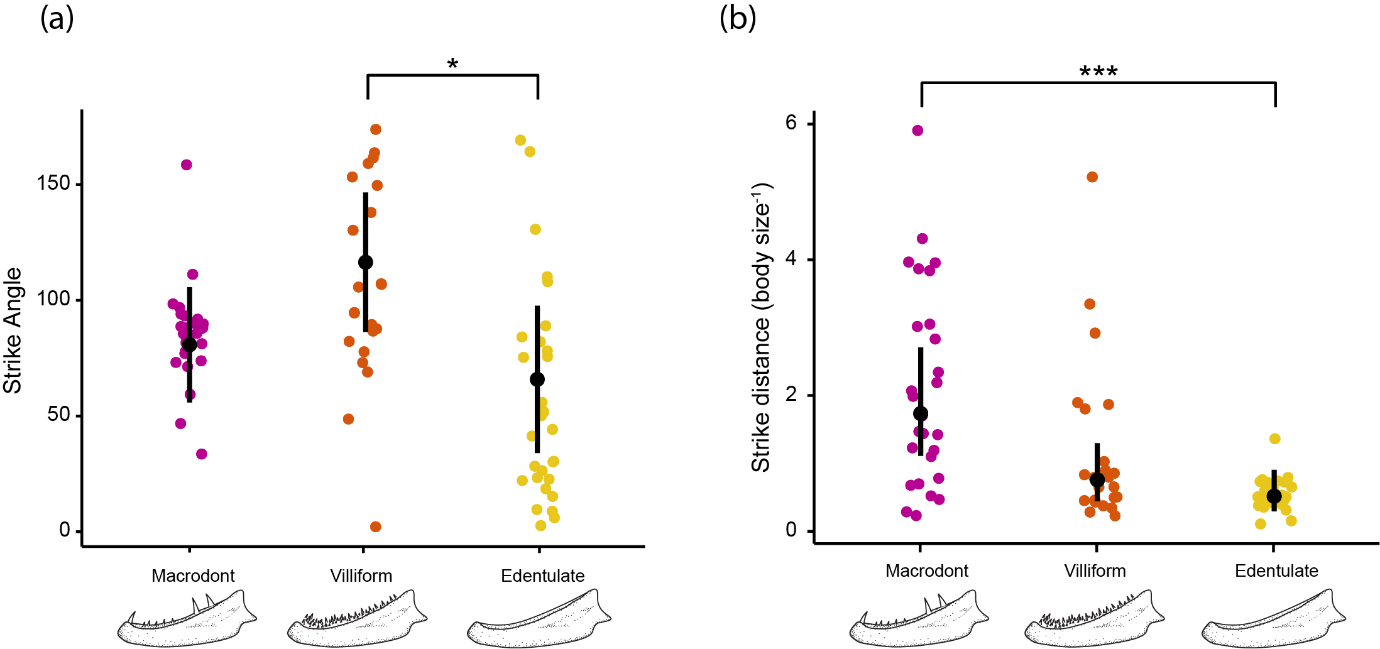


**Supplemental Figure 3.** (a) Strike angle and (b) distance of piscivorous fishes when striking at prey. Strike distance is shown relative to predators' body size (SL). Black bars represent means with 95% Confidence Intervals. Asterisks indicate level of significance between morphotypes.


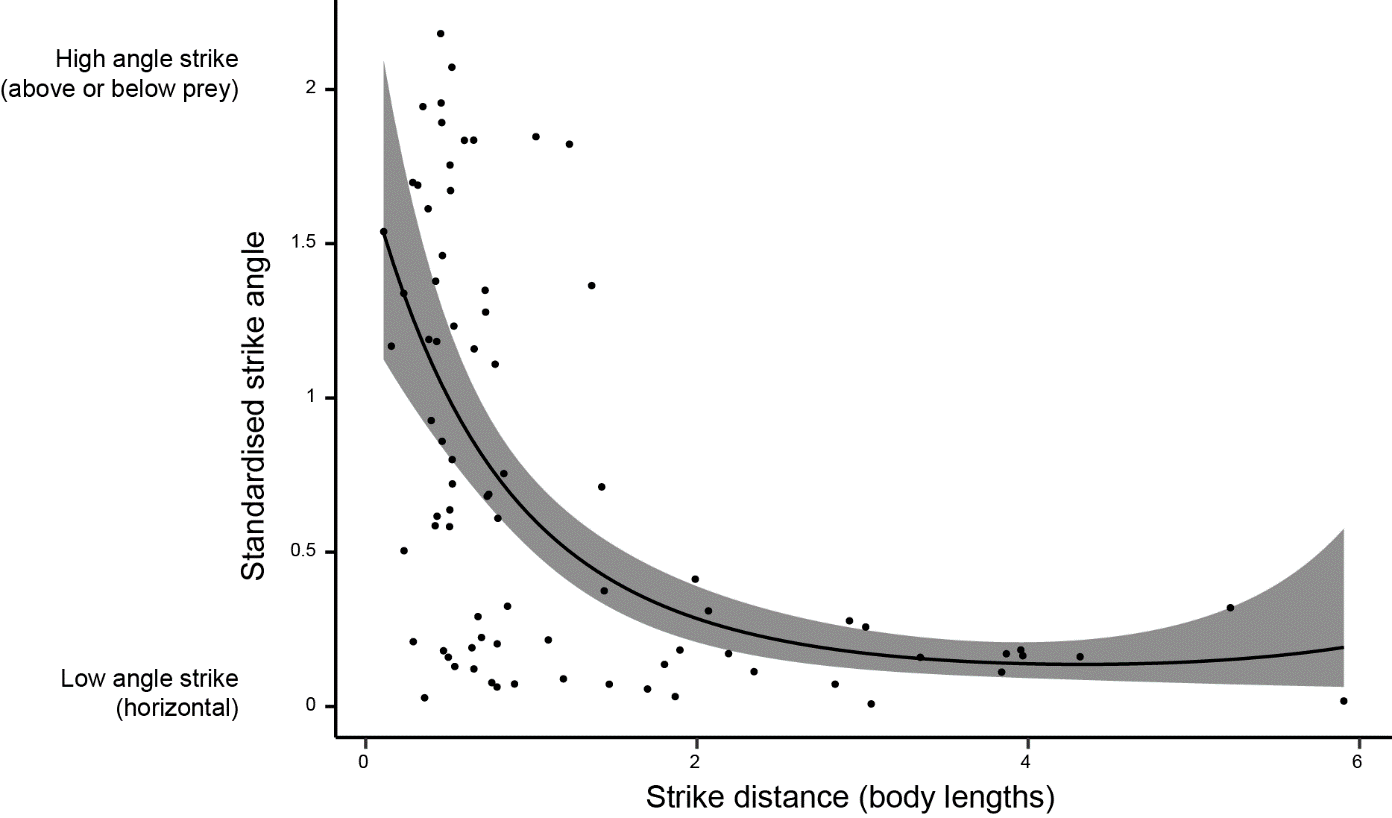


**Supplemental Figure 4**: Relationship between strike distance and absolute values of centred strike angle data, showing a significant (GLM; p<0.01) inverse relationship.


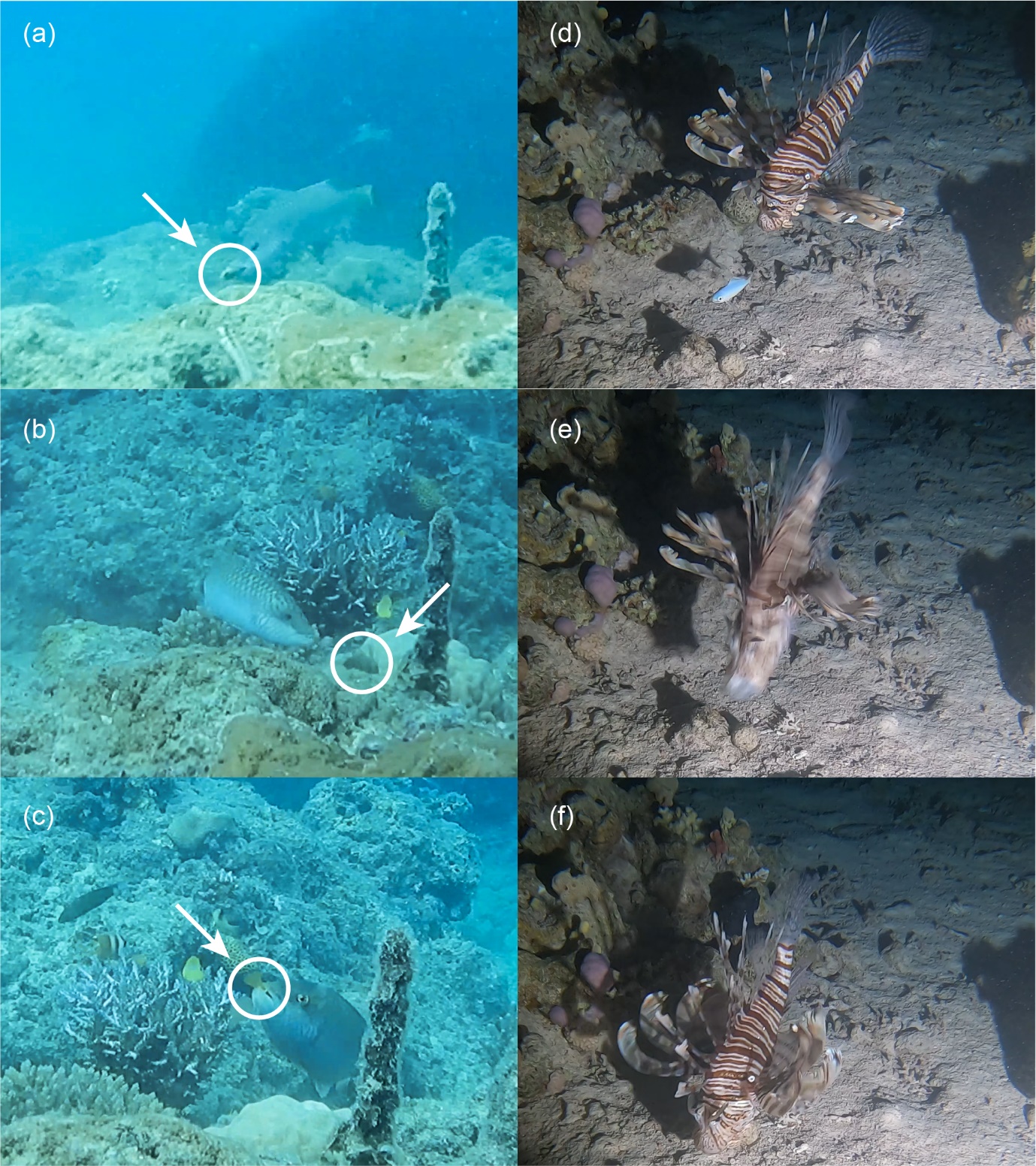


**Supplemental Figure 5:** Field-based predation events observed for the grabber *Oxycheilinus unifasciatus* (a-c), and the engulfer *Pterois volitans* (d-f). Upon tail-first capture (a) *O.unifasciatus* was observed by one of the authors (MM) to conduct headshaking behaviour (not captured in video footage), followed by prey *Pomacentrus sp.* being spat out (b), and re-ingested head-first (c). *P.volitans* is observed conducting an engulfing strike and capture. Credits for *P.volitans* footage: Salvatore Di Lauro.

**References**

Bellwood DR, Goatley CH, Bellwood O, Delbarre DJ, Friedman M (2015) The rise of jaw protrusion in spiny-rayed fishes closes the gap on elusive prey. Current Biology 25:2696-2700

Long JA (1991) Arthrodire predation by Onychodus (Pisces, Crossopterygii) from the Late Devonian Gogo Formation, Western Australia. Western Australian Museum: Records 15:503-516

Mihalitsis M, Bellwood DR (2019) Functional implications of dentition-based morphotypes in piscivorous fishes. Royal Society open science 6(9), 190040

Schaeffer B, Rosen DE (1961) Major adaptive levels in the evolution of the actinopterygian feeding mechanism. American Zoologist:187-204
